# Overexpression of secretory clusterin (sCLU) induces chemotherapy resistance in human gastric cancer cells by targeting miR-195-5p

**DOI:** 10.1080/21655979.2020.1747825

**Published:** 2020-04-06

**Authors:** Lihua Mu, Fengxia Yang, Dong Guo, Ping Li, Maoshen Zhang

**Affiliations:** a Department of General Surgery, The Affiliated Hospital of Qingdao University, Qingdao, Shandong, China; b Department of Ultrasound, The Affiliated Hospital of Qingdao University, Qingdao, Shandong, China

**Keywords:** Gastric cancer, chemoresistance, apoptosis, clusterin, miR-195-5p

## Abstract

Recent focus has turned to secretory clusterin (sCLU) as a key contributor to chemoresistance of anticancer agents, but the role of sCLU on chemotherapy drug response to gastric cancer cells is not fully understood. Previous research found that sCLU was overexpressed in the induced multidrug-resistant MGC-803/5-FU cell line, suggesting that sCLU upregulation was closely related to chemoresistance to anticancer agents. In the present study, we aimed to clarify the role and mechanisms of sCLU in regulating the chemoresistance of gastric cancer cells. Cell apoptosis and cell viability were evaluated by annexin V/propidium iodide staining and CCK8. Expression of sCLU and miR-195-5P was detected using quantitative RT-PCR assays. The expression of sCLU in gastric cancer tissues was detected by RT-PCR assays. Upregulating or downregulating sCLU or miR-195-5P in gastric cancer cells was used to evaluate the mechanisms of chemoresistance. We found that sCLU was significantly elevated in the MGC-803/5-FU and SGC-7901 cells, and the downregulating sCLU sensitized MGC-803/5-FU and SGC-7901 cells to cisplatin and Docetaxel by upregulation of miR-195-5P. Upregulating sCLU in MGC-803 and HGC-27 cells was resistant to cisplatin and Docetaxel by downregulating miR-195-5p. Targeting miR-195-5P reduced the sensitivity of MGC-803 cells to 5-FU, and miR-195-5P overexpression enhanced the sensitivity of MGC-803/5-FU cells to 5-FU. The overexpression of sCLU in gastric cancer tissues was associated with chemoresistance. Our findings suggest that overexpression of sCLU induced chemoresistance in gastric cancer cells by downregulating miR-195-5p, thus providing a potential target for the development of agents that targeting sCLU for gastric cancer therapy.

## Graphical Abstract

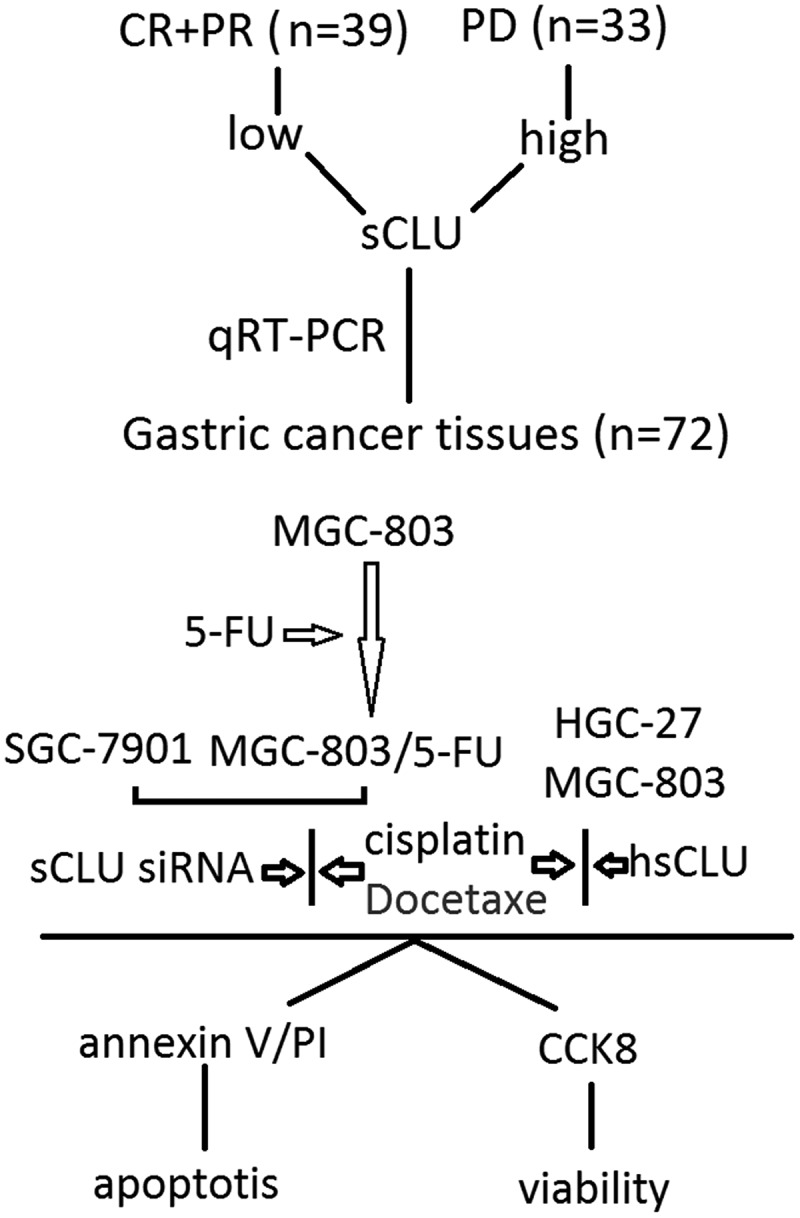

## Introduction

Gastric cancer (GC) is a critical health burden and the second common cause of cancer-related death globally [1]. Chemotherapy is the preferred treatment for advanced-stage GC patients. 5-Florouracil (5-FU)-based chemotherapy regimens, such as 5-FU in combination with cisplatin (DDP) or docetaxel, are generally accepted as the first-line treatments in advanced GC [2]. Despite the efficacy of chemotherapy for patients, the survival of patients with GC is substantially worse than that of patients with most other solid malignancies, and chemotherapy still achieves a limited response rate [3,4]. The mechanism of GC developing chemotherapy resistance is complicated and not well understood.

Clusterin (CLU), in its cytoplasmic secretory form (sCLU), has the unique property in mediating chemoresistance to numerous unrelated anticancer agents and its presence has been observed in a variety of solid tumors and lymphoma [5]. Miyake et al. [6] reported that *in vivo* administration of antisense sCLU oligonucleotides was demonstrated to significantly accelerate tumor regression and substantially delayed the development of androgen-independent tumors, indicating that sCLU is instrumental in acting as an antiapoptotic agent and facilitates survival and growth of tumors that no longer require androgen for their maintenance. Using these two tumor cell lines, sCLU was also implicated in the development of chemoresistance to gemcitabine [7]. Zellweger et al. [8] reported that pretreatment of Caki-2 human renal carcinoma cells with antisense-sCLU greatly enhanced chemosensitivity to paclitaxel *in vitro* and *in vivo* in nude mice. Using another model of breast cancer where taxanes are the established choice for management of metastatic disease, antisense-sCLU effectively chemosensitized MCF7 and MD-MB231 breast tumor cells to paclitaxel-induced apoptosis [9]. Thus, the potential of targeting sCLU in sensitizing tumor cells to chemotherapy has become an attractive new modality for cancer treatment.

It has recently reported that sCLU expression is associated with survival and an increase in disease recurrence in patients with colorectal cancer, and 5-FU resistance [10]. sCLU is found to be overexpressed in osteosarcoma (OS), and sCLU overexpression is associated with metastasis and chemoresistance. Furthermore, targeting sCLU inhibits metastasis and enhances chemosensitivity in OS cells [11]. Aberrant sCLU expression is involved in a number of molecular pathways related to the mechanisms of chemoresistance. A seminal report demonstrated that sCLU specifically binds activated Bax sequestering it from translocation to the mitochondria to induce cytochrome c release and apoptosis [12]. Others have corroborated this finding and demonstrated that sCLU binds and stabilizes the Ku70/Bax complex in the cytoplasm, retaining it as a complex and preventing its release of proteins that are potent in controlling the fate of a cell [13]. Additionally, a decrease in sCLU expression leading to AKT and ERK1/2 downexpression, resulting in chemosensitivity to DDP in A549 cells [14]. Emerging evidence showed that sCLU overexpression also plays an important role in tumor invasiveness [15]. Shiota et al. [16] demonstrated that sCLU is an important mediator of TGF-β-induced EMT, and suggest that sCLU sdownexpression may represent a promising therapeutic option for suppressing prostate cancer metastatic progression. However, Fischer et al. [17] reported that EMT does not affect lung metastasis development, but contributes to chemoresistance. This study suggests the potential of an EMT-targeting strategy by sCLU, in conjunction with conventional chemotherapies for GC treatment.

MicroRNAs are involved in regulating the biology of cancer cells, the recent discovery of miRNAs in malignancy has provided new directions for research on mechanisms underlying response to chemotherapy [18]. Furthermore, several studies have documented that selected miRNAs, such as miR-137 [19], miR-223 [20], miR-126-5P [21], miR-221 [22] and miR-33a [23] may influence chemotherapy response in several tumor types, including gastric cancer. miR-195-5P downexpression has found to be associated with poor prognosis and poor chemotherapy response to drugs in breast cancer [24]. In colorectal cancer (CRC) cells, enforced miR-195-5p significantly increased tumor cell apoptosis and decreased tumor sphere formation as well as reduced cell stemness and chemoresistance [25]. Alacam et al. [26] has investigated the relation of miRs to treatment resistance in schizophrenic patients, and found that miR-195-5p expression was significantly different between the drug-resistant groups and drug-sensitive groups, which was higher in miR-195-5p expression. In GC cells, enforced miR-195-5p inhibits GC tumorigenesis *in vivo* through suppressing bFGF [27], and dysregulation of miR-195-5p/-218-5p/BIRC5 axis predicts a poor prognosis in patients with GC [28].

In this study, a multiple-drug-resistant cell line MGC-803/5-FU was derived from MGC-803 cells by exposing them to gradually increasing concentrations of 5-FU. We found that sCLU was up-regulated in the MGC-803/5-FU cells and sCLU up-regulation was correlated with chemoresistance; sCLU expression was also significantly higher in chemoresistant GC tissue. Mechanically, we confirmed that miR-195-5P was regulated by sCLU, and sCLU induces chemoresistance in human GC cells by miR-195-5P downexpression.

## Methods

### Cell lines and culture conditions

The GC cell lines MGC-803, HGC-27, BCG-823, MKN-28, MKN-45, and SGC-7901 were obtained from Shanghai Institute for Biological Sciences, Chinese Academy of Science. MGC-803-derived 5-FU-resistant sublines (MGC-803/5-FU) were induced by gradual exposure of 5-FU in culture medium. Briefly, MGC-803 cells were cultured in fresh medium without drugs for 24 h. Subsequently, the medium was changed and 0.01 μM 5-FU in complete medium was added. MGC-803 cells were exposed to 5-FU for 48–72 h; thereafter, the 5-FU-treatment medium was removed and cells were allowed to recover (in normal medium) for about 1 week. When cells reached 70% confluence, the treatment process was repeated for several times until they were stable (3–4 weeks). Once stable, cells were subjected 0.01, 0.1, 0.6 and finally 1.2 μM 5-FU treatment. Thereafter, the 5-FU-resistant cells were maintained in full medium supplemented with 1.2 μM 5-FU (to maintain 5-FU resistance). All GC cell lines were maintained at 37°C in a humidified atmosphere of 5% CO_2_ and 95% air in RPMI 1640 medium (Thermo Electron Corporation, Beijing, China) supplemented with 10% (v/v) fetal bovine serum (FBS; Life Tech, Mulgrave Vic, Australia) and penicillin/streptomycin (10,000 IU/ml penicillin and 20 mg/ml streptomycin; Roche, Swiss). The medium was changed twice per week.

### siRNA and plasmid preparation

The small interfering RNAs (siRNAs) corresponding to human sCLU mRNA sequences 5′-AGGAAGAACCCUAAAUUUA-3′, nucleotide positions 1578–1596 in clusterin mRNA (sCLU siRNA) and non-human siRNA 5′-GUCCGGGCAGGUCUACUUU-3′ was purchased from Shanghai GenePharma Co. Ltd., Shanghai, China. The full-length human sCLU cDNA clone was initially obtained from the American Type Culture Collection (Manassas, VA). Human sCLU was amplified using TaKaRa LA Taq and cloned into the pIRES2-ZsGreen1 vector (CLONTECH), which was named pIRES2-ZsGreen1-hsCLU (hsCLU). The pIRES2-ZsGreen1 vector (vector) was as the control.

### Cell transfection

An hsCLU (50 nM) was transfected into MGC-803, HGC-27 cells, and hsCLU siRNA (50 nM) was transfected into MGC-803/5-FU and SGC-7901 cells using Lipofectamine 2000 (Invitrogen) in serum-free DMEM. Transfection complexes were prepared according to the manufacturer’s instructions (GenePharma Co., Shanghai, China).

miR-195-5p inhibitor, miR-195-5p mimic, negative control (NC) were purchased from RiboBio (Guangzhou, China). MGC-803 cells were transfected with miR-195-5p inhibitor (50 nM) or NC-inhibitor (50 nM); MGC-803/5-FU cells were transfected with miR-195-5p mimic (50 nM) or NC- mimic (50 nM) using Lipofectamine 2000 (Invitrogen) in serum-free DMEM.

### Measurement of cell viability by cholecystokinin octapeptide (CCK8) assay

All cells were seeded in 96-well plates at a density of 2 × 10^4^ cells/well. After transfection with sCLU siRNA for 12 h, 5-FU, cisplatin and Docetaxel were added at different concentrations, and after growth for 72 h, 10 ul of the CCK8 reagent were added to each well. The plates were incubated for 4 h at 37°C, and the difference in absorbance between 450 and 590 nm was measured as an indicator of cell viability.

In addition, after transfection with miR-195-5P mimic or miR-195-5P inhibitor for 12 h in MGC-803/5-FU or MGC-803 cells, 5-FU was added at concentration 0.12 μM, and after growth for 72 h, 10 μl of the CCK8 reagent were added to each well. The plates were incubated for 4 h at 37°C, and the difference in absorbance between 450 and 590 nm was measured as an indicator of cell viability.

### Apoptosis analysis by annexin V/Propidium iodide (PI) staining

Apoptotic and dead cell counts were performed using FITC-labeled annexin V and PI staining (BD Biosciences), followed by flow cytometry. The cells were gently vortexed and resuspended in binding buffer at a concentration of 3 x10^6^/ml, and then 100 μl of cell suspension was added to 5 μl of annexin V-FITC and 10 μl of PI and mixed for 15 min in the dark at room temperature. Next, 400 μl of PBS was added to the solution. A FACScan instrument (BD Biosciences) was used to count the cells (1x10^3^) at an excitation wavelength of 490 nm. CellQuest software was used for data collection and processing.

### Patients and specimens

This study included 178 patients with GC that had invaded into submucosal layer or deeper. All patients underwent curative gastrectomy with lymph node dissection at the affiliated hospital of Qingdao University from February 2015 to December 2018. Among 178 patients, 58, 15, 99, and 5 patients received distal, proximal, total and partial gastrectomy, respectively. The male to female ratio was 117 to 60, and the average age was 65.7 years, ranging from 32 to 78 years. The number of patients with stage I, II, and III gastric cancers was 49, 30, and 99, respectively. Histopathologically, 71 cases were differentiated (papillary, well-differentiated and moderately differentiated tubular adenocarcinoma) and 106 were undifferentiated (poorly differentiated adenocarcinoma, mucinous adenocarcinoma, and signet-ring cell carcinoma) carcinomas according to the 7^th^ edition of tumor node metastasis classification. None of these patients received chemotherapy before surgery. Chemotherapeutic efficacy was evaluated after two treatment cycles according to the standard of RESIST version 1.1, as four outcome measures, including complete response (CR), partial response (PR), stable disease (SD), and progressive disease (PD). Patients with efficacy assessments of CR+PR were categorized as having effective chemotherapy outcomes, and patients with results of PD were categorized as having ineffective treatment outcomes. CR+PR was used to calculate the objective response rate (ORR), and CR+PR+SD was used to calculate the disease control rate (DCR). Written informed consent was obtained from the patients and the study was approved by the Ethical Committee of the affiliated hospital of Qingdao University. This investigation conformed to the principles outlined in the Declaration of Helsinki. Clinicopathological findings are described according to the 7^th^ edition of tumor node metastasis classification.

### RNA isolation and real-time qRT-PCR

Total RNA was extracted from tissues and cells using TRIzol reagent (Invitrogen) and was reverse transcribed using an M-MLV RT Kit (Promega). For the detection of mature miR-195-5p, sCLU specific RT primers and PCR primers (Gene Copoeia) were constructed, and cDNA was amplified using SYBR® Green PCR Master Mix (Toyobo, Osaka, Japan). Relative expression was normalized to snRNA U6 (U6)/GAPDH expression and was calculated using the 2-ΔΔCt method.

### Association between the expression sCLU in gastric cancer and chemoresistance

To evaluate whether the expression level of sCLU in gastric cancer affects chemoresistance, we compared the expression level of sCLU in gastric cancer patients who received 5-fluorouracil (5FU) based adjuvant chemotherapy. We defined ‘5FU resistance’ as gastric cancer that recurred after adjuvant chemotherapy. As a standard protocol, we administer 5FU-based adjuvant chemotherapy to patients with stage II or III cancer. We excluded patients who were not able to continue adjuvant chemotherapy due to side effects. As a result, 72 patients were enrolled in this analysis.

### Statistical analysis

The significance of differences was determined by the statistical software GraphPad prism for windows. T-test, one-way or two-way ANOVA was used adequately when needed and followed by Tukey's *post-hoc* multiple comparison test. All results are shown as mean and the standard deviation (mean ± SD). *p* < 0.05 was considered significant.

## Results

### The establishment of the 5-Fu-resistant cell line MGC-803

**Figure 1.**
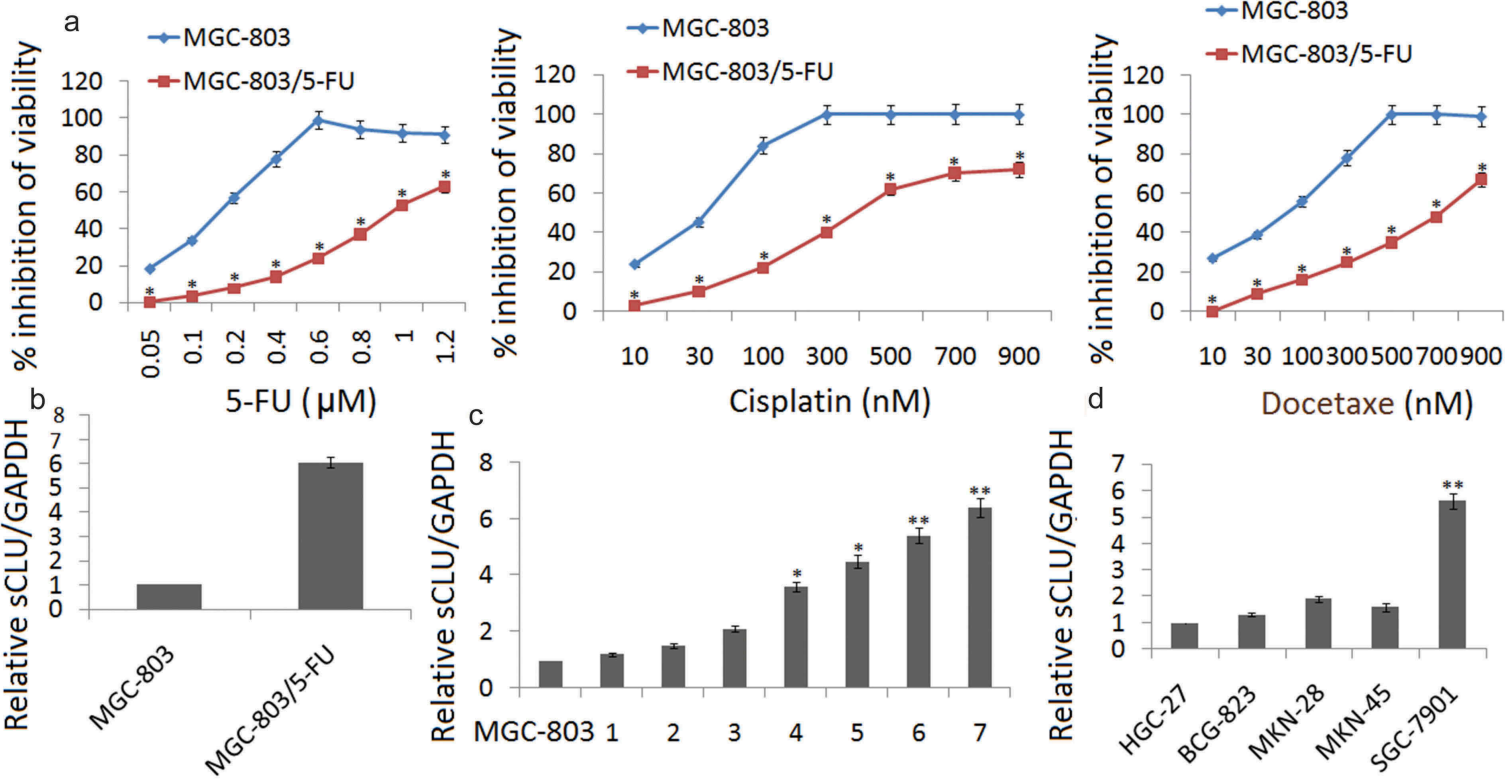
The up-regulation of sCLU expression in chemoresistant gastric cancer cell lines. (a) Establishment of the 5-FU-resistant MGC-803 (MGC-803/5-FU) cell lines. The viability of MGC-803 and MGC-803/5-FU cells at various concentrations of cisplatin and Docetaxel was determined by a CCK8 assay (*p < 0.05 compared with MGC-803). (b) sCLU expression is up-regulated in MGC-803/5-FU cells by qRT-PCR assay. (c) qRT-PCR for sCLU shows that sCLU expression is the lowest in MGC-803 cells. The x axis labels (1–7) represent the different generations of MGC-803/5-FU selection. sCLU expression was dramatically increased, by more than 5-fold, in the seventh generation of MGC-803/5-FU compared with MGC-803 (*p < 0.05; ** *p* < 0.01 compared with the MGC-803 group). (d) sCLU expression is up-regulated in SGC-7901 cells. sCLU expression detected by qRT-PCR is the lowest in HGC-27 cells. The level of sCLU expression in SGC-7901 was almost 5-fold higher than that in HGC-27 cells (**p < 0.01 compared with HGC-27).

Over a period of 6 months, MGC-803 cells in culture were continuously exposed to increasing concentrations of 5-FU. As shown in Figure 1(a), the IC50 of 5-FU in the MGC-803 cell line was 0.12 μM, and the exposure dose was progressively increased. The IC50 of 5-FU in the established MGC-803/5-FU was 0.98 μM. The sensitivities of MGC-803 and MGC-803/5-FU cells to various concentrations of cisplatin and Docetaxel were determined by the CCK8 assay. The IC50 values for cisplatin and docetaxel in MGC-803 cells were 30.43 and 90.08 nM, respectively, 323.26 nM and 714.53 nM in MGC-803/5-FU cells, respectively. The MGC-803/5-FU cell line acquired resistance to not only 5-FU, but also cisplatin and docetaxel. Taken together, these data indicate that MGC-803/5-FU is a multiple-drug-resistant cell line.

### sCLU is up-regulated in MGC-803/5-FU cells

qRT-PCR assay was used to detect sCLU expression in MGC-803 and MGC-803/5-FU cells. The results showed that sCLU expression was significantly upregulated in the MGC-803/5-FU cells compared with the expression of sCLU in MGC-803 cells (Figure 1(b)). We then measured the sCLU expression in the different generations of MGC-803/5-FU using qRT-PCR. sCLU expression was dramatically increased more than 6-fold in the seventh generation of MGC-803/5-FU cells compared with the MGC-803 cells, implying that sCLU increased sharply under stringent drug stress and was related to drug sensitivity (Figure 1(c)). In addition, we also measured the sCLU expression in other gastric tumor cell lines, including HGC-27, BCG-823, MKN-28, MKN-45 and SGC-7901 cells. Because the level of sCLU in SGC-7901 was almost 5-fold higher than that in HGC-27 cells, we chose this pair of cell lines, SGC-7901 and HGC-27, to confirm our hypothesis (Figure 1(d)).

### sCLU modulated the chemoresistance of human gastric cancer cells

**Figure 2.**
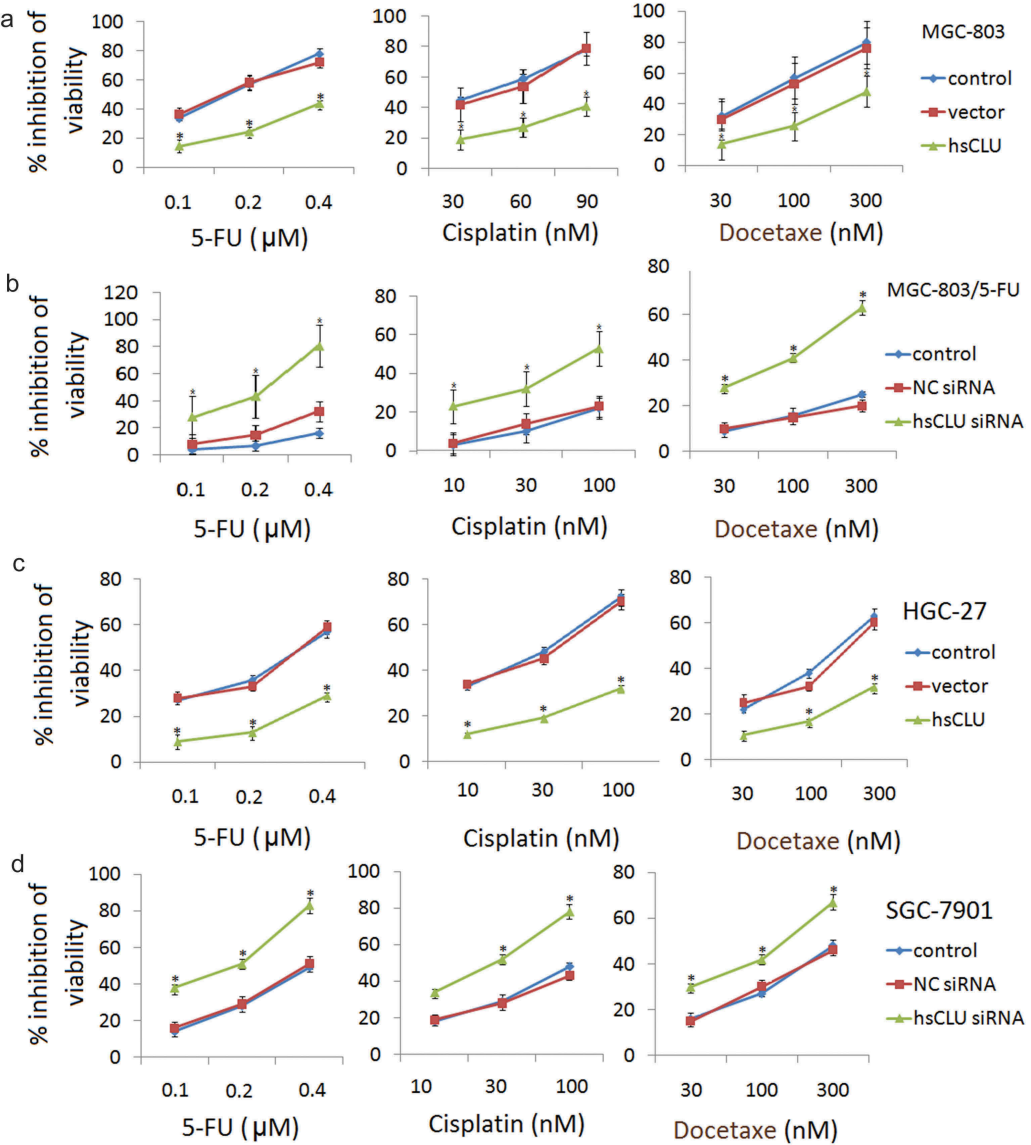
sCLU modulates the resistance of human gastric cancer cells to chemotherapeutic drugs. Following transfection with either the negative control RNA (vector) or hsCLU, MGC-803 cells and HGC-27 cells were treated with a series of concentrations of 5-FU, cisplatin, and docetaxel, respectively. The effect of the hsCLU on the viability of MGC-803 cells (a) and HGC-27 cells (c) in response to these three chemotherapeutic drugs was determined using the CCK8 assay. *p < 0.05 compared with the control group. Following either the negative control RNA (NC siRNA) or sCLU siRNA, MGC-803/5-FU cells and SGC-7901 cells were treated with a series of concentrations of 5-FU, cisplatin and docetaxel. The effect of sCLU siRNA on the viability of MGC-803/5-FU (b) and SGC-7901 cells (d) in response to these three chemotherapeutic drugs was determined using the CCK8 assay. *p < 0.05 compared with the control group.

We further investigated whether inhibiting or increasing sCLU expression could modulate cell survival and the sensitivity of MGC-803 and MGC-803/5-FU cells to chemotherapeutic drugs, including 5-FU, cisplatin, and docetaxel, all of which are currently used for the treatment of GC. Following transfection of the hsCLU into MGC-803 cells, we treated the cells with a series of concentrations of 5-FU, cisplatin, and docetaxel. The effect of hsCLU on the chemoresistance of MGC-803 cells is shown in Figure 2(a)) These results imply that the introduction of hsCLU notably reduced the chemosensitivity of MGC-803 cells to 5-FU, cisplatin, and docetaxel. In addition, the inhibition of MGC-803/5-FU cell growth by the chemotherapeutic drugs was significantly increased by transfection with sCLU siRNA (Figure 2(b)). To further investigate the function of sCLU, we also examined the toxicity of 5-FU, cisplatin, and docetaxel in SGC-7901 and HGC-27 cells, in which sCLU was differentially expressed. HGC-27 cells expressing hsCLU increased viability in response to treatment with 5-FU, cisplatin, and docetaxel compared with the control group (Figure 2(c)), whereas SGC-7901 cells transfected with sCLU siRNA exhibited significantly decreased viability in response to chemotherapeutic drugs (Figure 2(d)).

### sCLU regulates miR-195-5p and apoptosis in human gastric cancer cells

**Figure 3.**
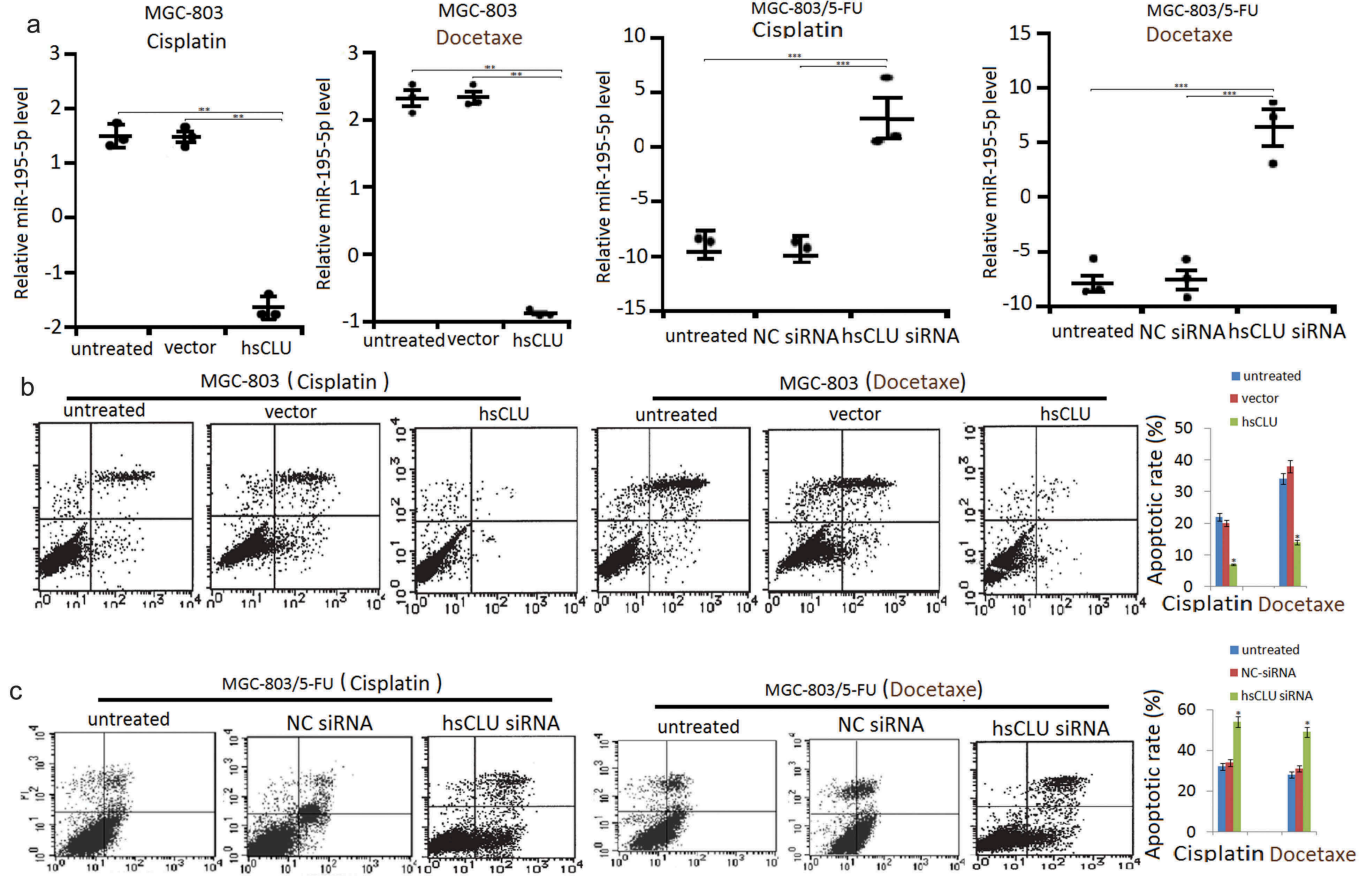
sCLU regulates miR-195-5p level and apoptosis in human gastric cancer cells. (a) MGC-803 cells were transfected with hsCLU and then treated with cisplatin or docetaxel. MGC-803/5-FU cells were transfected with hsCLU siRNA and then treated with cisplatin or docetaxel. The miR-195-5p levels of MGC-803 cells and MGC-803/5-FU cells were determined by qRT-PCR (a), and apoptotic rate in MGC-803 cells were detected by annexin V/PI (b) (*p < 0.05, ** *p* < 0.01, *** *p* < 0.001, compared with the untransfected group), and apoptotic rate in MGC-803/5-FU cells was detected by annexin V/PI (c) (*p < 0.05, compared with the untransfected group).

To explore the effects of sCLU on cell apoptosis, MGC-803 cells were transfected with hsCLU and subsequently treated with cisplatin (60 nM) and docetaxel (100 nM). In parallel, MGC-803/5-FU cells were transfected with sCLU siRNA and then treated with cisplatin (100 nM) and docetaxel (300 nM). The cells were collected to assess the miR-195-5p levels by qRT-PCR and the apoptosis ratios by annexin V/PI staining. The results demonstrated that sCLU siRNA induced cell apoptosis, accompanied by an increase of miR-195-5p level in the cells (Figure 3(a,b). We also detected the effects of the sCLU and of sCLU siRNA on cell apoptosis and the miR-195-5p level in HGC-27 cells and SGC-7901 cells, respectively, upon treatment with cisplatin or docetaxel. Transfection with an sCLU notably decreased the cell apoptotic ratio and miR-195-5p levels of HGC-27 cells undergoing treatment with cisplatin or docetaxel (data not shown) compared with the control group, whereas transfection with sCLU siRNA increased the apoptotic ratio and miR-195-5p levels of SGC-7901 cells (data not shown) undergoing treatment with chemotherapeutic drugs. We further detected a change in apoptosis-related proteins after treatment of MGC-803 with the sCLU.

### sCLU regulates the chemosensitivity of human gastric cancer cells by targeting miR-195-5p

**Figure 4.**
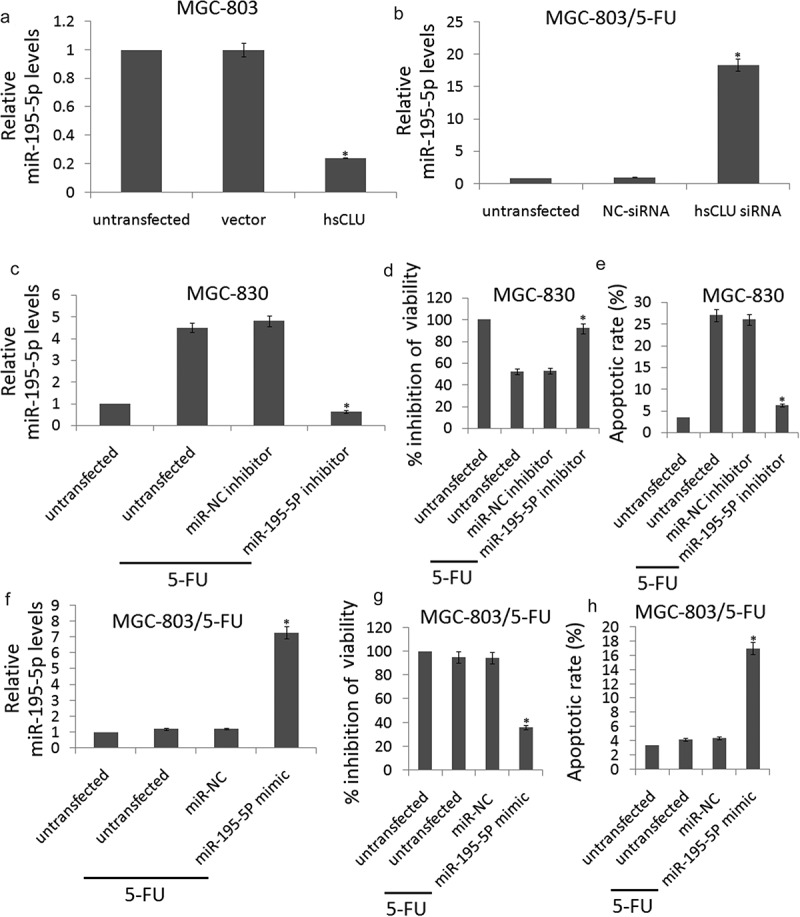
sCLU regulates the chemosensitivity of gastric cancer cells by targeting miR-195-5P.(a, b), miR-195-5P levels in MGC-803 cells transfected with hsCLU and MGC-803/5-FU cells transfected with sCLU siRNA were verified by qRT-PCR (*p < 0.01) compared with the control group). (c) qRT-PCR for miR-195-5P expression in MGC-803 cells that were untransfected or transfected with miR-NC inhibitor or miR-195-5P inhibitor (*p < 0.01 compared with the untransfected group). (d) The effect of miR-195-5P inhibitor on the viability of MGC-803 cells during 5-FU treatment was determined using the CCK8 assay (*p < 0.01 compared with the untransfected group). (e) The effect of miR-195-5P inhibitor on apoptosis in MGC-803 cells undergoing 5-FU treatment was determined by annexin V/PI staining, respectively (*p < 0.01 compared with the untransfected group). (f) qRT-PCR for miR-195-5P expression in MGC-803/5-FU cells that were untransfected or transfected with miR-NC or miR-195-5P mimic (*p < 0.01 compared with the untransfected group). (g) the effect of miR-195-5P mimic on the viability of MGC-803/5-FU cells during 5-FU treatment was determined using the CCK8 assay (*p < 0.01 compared with the untransfected group). (h) The effect of miR-195-5P mimic on apoptosis in MGC-803/5-FU cells undergoing 5-FU treatment was determined by annexin V/PI staining, respectively (*p < 0.01 compared with the untransfected group).

To further confirm the potential role of sCLU in the regulation of miR-195-5p, we evaluated the miR-195-5p expression levels in MGC-803 and MGC-803/5-FU cells in which the sCLU was introduced or sCLU siRNA treatment had suppressed the endogenous sCLU. As shown in Figure 4(a,b), the up-regulation of sCLU by the hsCLU transfection led to a notable decrease in the miR-195-5p levels, whereas sCLU siRNA transfection up-regulated the miR-195-5p levels. This inverse correlation between sCLU and miR-195-5p levels provided sufficient evidence to support our conclusions. The data demonstrated that miR-195-5p was a target of sCLU in GC cells.

To elucidate the functional contributions of miR-195-5p to the chemosensitivity of GC cells, we transfected MGC-803 cells with miR-195-5p inhibitor to targeting miR-195-5p. As shown in Figure 4(c), the miR-195-5p inhibitor significantly inhibited miR-195-5p in the MGC-803 cells in response to treatment with 5-FU (0.12 μM). We examined the viability and apoptosis of miR-195-5p inhibitor-transfected MGC-803 cells in response to treatment with 5-FU (0.12 μM). The CCK8 assay and apoptosis analysis showed that the chemoresistance of MGC-803 cells increased upon miR-195-5p knockdown with miR-195-5p inhibitor (Figure 4(d,e)).

We next transfected MGC-803/5-FU cells with miR-195-5p mimic to increase miR-195-5p expression. As shown in figure 4(f), the miR-195-5p mimic significantly increased miR-195-5p levels in the MGC-803/5-FU cells in response to treatment with 5-FU (0.12 μM). We examined the cell viability and apoptosis of miR-195-5p mimic-transfected MGC-803/5-FU cells in response to treatment with 5-FU (0.12 μM). The CCK8 assay and apoptosis analysis showed that the chemosensitivity of MGC-803/5-FU cells increased upon miR-195-5p overexpression with miR-195-5p mimic (Figure 4(g,h)).

### sCLU level in gastric cancer and its association with 5-FU resistance

**Figure 5.**
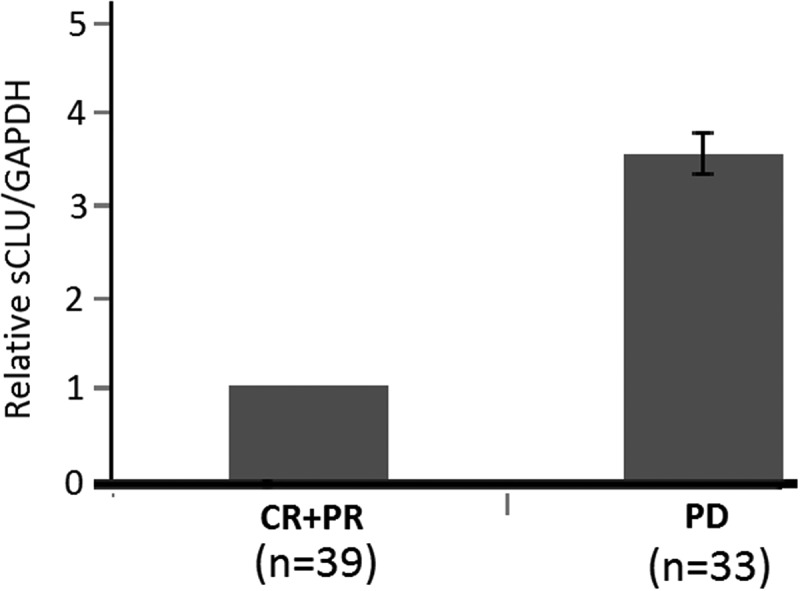
Expression of sCLU and treatment efficacy in patients with gastric cancer. There is a significant difference in patients with effective and ineffective treatment outcome (*p* < 0.01, unpaired t-test).

Among those 72 patients, 16 had effective treatment outcomes and 33 patients had ineffective treatment outcomes and 23 patients were in stable condition. Patients with efficacy assessments of CR+PR (n = 39) were categorized as having effective chemotherapy outcomes, and patients with results of PD (n = 33) were categorized as having ineffective treatment outcomes. As shown in Figure 5, relative sCLU expression for patients with effective chemotherapy outcomes (CR+PR) was significantly lower than for patients with ineffective outcomes (PD) (*p* < 0.01).

## Discussion

Individualized management for tumor patients is one of the most important therapeutic principles. The implementation of adjuvant therapy and chemotherapy has had a major impact on the disease-free survival and overall survival of gastric cancer patients, but the median survival time is often less than 1 year and none of the regimens has yet emerged as a clear standard [29,30]. However, most GC patients have advanced or metastatic diseases at diagnosis [31]. While surgical resection is an effective therapeutic procedure for curing gastric cancer patients, the 5-year survival rate is only about 20% for patients with late-stage GC [29]. Therefore, early diagnosis is beneficial and critical for successful surgical removal of GCs since peritoneal dissemination and local/distal metastases often occur in the late stages of GC and greatly reduce the effectiveness of surgery intervention. Unfortunately, early GC diagnosis is not feasible for most GC patients because of the endoscopic gastroscopy that is required to confirm the diagnosis and the lack of useful convenient noninvasive detection biomarkers for routine population screening. In recent years, there have been advancements in the molecular biomarkers utilized in cancer detection and in the development of therapeutic agents based on the target genes for a few types of solid tumors excluding gastric cancer [30]. Useful diagnostic biomarkers for early gastric cancer detection remain limited; therefore, it is essential to devote more research to investigate these biomarkers in the near future.

Clusterin (CLU) is a key contributor to chemoresistance to anticancer agents, and only the cytoplasmic/secretory clusterin form (sCLU) is expressed in aggressive late-stage tumors, which is in line with its antiapoptotic function [32–36]. Most significantly, sCLU expression is documented to lead to broad-based resistance to other unrelated chemotherapeutic agents such as doxorubicin, cisplatin, etoposide, and camphothecin [37–39]. However, the role of sCLU in gastric cancer, especially its correlation with chemosensitivity, has been unclear. In this paper, we identified sCLU was up-regulated in MGC-803/5-FU cells compared with MGC-803 cells. By modulating the sCLU level in GC cells, we revealed that sCLU mediated the chemoresistance of GC cells through the anti-apoptosis pathway. These observations were extended to clinical samples of GC, where we found a positive correlation between sCLU levels and chemoresistance.

In this study, MGC-803 cells display marked resistance when sCLU expression was increased, and vice versa. This may be due to the following aspects: The increased expression levels of sCLU may directly translate to elevated drug resistance, and sCLU may influence the expression of certain other genes and may be associated with the response to other drugs. For instance, various other studies have reported that sCLU was associated with drug resistance of osteosarcoma cells [11] and prostate cancer cells [40]. Furthermore, the increased resistance to drugs was not entirely due to the elevation of sCLU, and is probably regulated by additional signaling pathways, including the activating AKT/GSK3β/β-catenin axis [41] and Wnt/β-catenin and IGF-I signaling [40]. All of the above suggested that the mechanism of drug resistance was highly complex, while it is indicated that sCLU may have an important role in the multidrug resistance of cancer cells.

It is broadly accepted that certain miRNAs regulate a large number of protein-coding genes during cell apoptosis. miR-195-5p acts as a strong pro-apoptotic role and tumor-suppressive role in many cancers, such as human colorectal cancer [42], non-small cell lung cancer cells [43], prostate cancer [35] and gastric cancer [28]. miR-195-5p -mediated apoptosis in tumors is regulated by numerous canonical signaling pathways, among which induces the expression of anti-apoptotic Bcl-2, and phosphorylates and inactivates the pro-apoptotic Bcl-2 family member Bcl-2-associated death promoter, which induces the expression of genes that are critical for cell death. The present results indicated that sCLU regulates drug resistance and cell apoptosis in GC by targeting the tumor suppressor miR-195-5p. In future studies, specific knockdown or inhibition of individual pathways may provide a deeper understanding of these interactions.

The present study is limited by its lack of animal experiments, which would have provided more powerful evidence for implementation of the results in drug therapy. However, the present study employed various experimental methods and combined clinical data for analysis in the present study. A future study by our group will assess the function of sCLU in a mouse model.

Our findings from cell lines and clinical samples suggest that the overexpression of sCLU induced chemoresistance in GC cells by downregulating miR-195-5p. sCLU was overexpressed in chemoresistant GC tissues but reduced in chemosensitive tissues, and on the contrary, miR-195-5p expression was higher in chemosensitive than in chemoresistant GC tissues. This inverse correlation between sCLU and miR-195-5p levels provided sufficient evidence to indicate that miR-195-5p was a target of sCLU in GC cells.

## Conclusion

In summary, we identified the function, target, and upstream regulatory mechanism of sCLU, both in cell lines and in clinical samples, providing evidence that sCLU targets miR-195-5p to mediate the chemotherapeutic resistance of GC cells. We extended the current knowledge by highlighting the role of sCLU in the chemoresistance of GC for the first time. Although chemotherapy is the backbone of systemic treatment for most malignancies, its efficacy is hindered by the development of drug resistance. Therefore, targeting the mechanisms involved in the chemoresistance of GC will improve treatment efficacy. It is logical to predict that sCLU could be a potential biomarker and that the inhibition of sCLU may be a candidate strategy for new GC therapies.

## Highlights

1. Overexpression of sCLU was related to chemotherapeutic resistance in gastric cancer tissues and cell lines
2. Targeting sCLU enhanced the chemosensitivity of gastric cancer cells *in vitro*, and vice versa.
3. sCLU targets miR-195-5p to mediate the chemotherapeutic resistance of gastric cancer cells.
4. sCLU could be a potential biomarker for predicting chemosensitivity in gastric cancer.
